# Ultradeep Sequencing of a Human Ultraconserved Region Reveals Somatic and Constitutional Genomic Instability

**DOI:** 10.1371/journal.pbio.1000275

**Published:** 2010-01-05

**Authors:** Anna De Grassi, Cinzia Segala, Fabio Iannelli, Sara Volorio, Lucio Bertario, Paolo Radice, Loris Bernard, Francesca D. Ciccarelli

**Affiliations:** 1 Department of Experimental Oncology, European Institute of Oncology, Milan, Italy; 2 IFOM, Fondazione Istituto FIRC di Oncologia Molecolare, IFOM-IEO Campus, Milan, Italy; 3 Hereditary Colorectal Tumor Registry; Fondazione IRCCS Istituto Nazionale dei Tumori, Milan, Italy; 4 Department of Experimental Oncology, Fondazione IRCCS Istituto Nazionale dei Tumori, Milan, Italy; Medical Research Council Human Genetics Unit, United Kingdom

## Abstract

Early detection of cancer-associated genomic instability is crucial, particularly in tumour types in which this instability represents the essential underlying mechanism of tumourigenesis. Currently used methods require the presence of already established neoplastic cells because they only detect clonal mutations. In principle, parallel sequencing of single DNA filaments could reveal the early phases of tumour initiation by detecting low-frequency mutations, provided an adequate depth of coverage and an effective control of the experimental error. We applied ultradeep sequencing to estimate the genomic instability of individuals with hereditary non-polyposis colorectal cancer (HNPCC). To overcome the experimental error, we used an ultraconserved region (UCR) of the human genome as an internal control. By comparing the mutability outside and inside the UCR, we observed a tendency of the ultraconserved element to accumulate significantly fewer mutations than the flanking segments in both neoplastic and nonneoplastic HNPCC samples. No difference between the two regions was detectable in cells from healthy donors, indicating that all three HNPCC samples have mutation rates higher than the healthy genome. This is the first, to our knowledge, direct evidence of an intrinsic genomic instability of individuals with heterozygous mutations in mismatch repair genes, and constitutes the proof of principle for the development of a more sensitive molecular assay of genomic instability.

## Abstract 2

Ultradeep sequencing of genomes permits the detection of very low-level genomic instability in non-neoplastic tissues of patients with the most common form of inherited colorectal cancer.

## Author Summary

In hereditary non-polyposis colorectal cancer (HNPCC), a germline mutation in one allele of a gene responsible for repairing DNA damage predisposes the host to cancer, because subsequent somatic inactivation of the one wild-type allele leads to genomic instability that favours tumourigenesis. Nonneoplastic tissues of HNPCC individuals are believed to repair DNA normally, as they are heterozygous and thus are thought to be genomically stable. However, methods used to date are known to be incapable of detecting very low levels of genome instability. Here, we present a more sensitive procedure based on the resequencing of a HNPCC genomic region using next-generation sequencing technology. With this approach, we show that genomic instability is in fact detectable in nonneoplastic tissues of HNPCC patients compared with healthy donors. This constitutional instability may predispose them to acquiring the second somatic mutation event needed for cancer development.

## Introduction

Genomic instability is a common trait of cancer cells and plays a pivotal role in promoting carcinogenesis in several hereditary tumours. One of the best-known examples is the Lynch syndrome, an autosomal dominant condition associated with heterozygous mutations in mismatch repair (MMR) genes [1]. During their lifespan, individuals affected by the Lynch syndrome undergo somatic inactivation of the second allele that causes the impairment of the MMR machinery and the onset of the “mutator phenotype” [2]. The tumourigenic process starts when mutations hit oncogenes and/or tumour suppressors, often in actively renovating tissues such as endometrium, ovary, and colon. In the latter case, the genetic condition is known as hereditary non-polyposis colorectal cancer (HNPCC), which represents the most common form of inherited colorectal cancer [3]. A hallmark of MMR deficiency is microsatellite instability (MSI), which measures the accumulation of insertions and deletions (indels) at repeated regions of the genome. Since more than 90% of HNPCC show MSI [4],[5], this has become a common diagnostic marker of MMR deficiency. Recently, large-scale mutational screenings returned the first estimations of the mutation frequency, which is the number of mutations per genome unit, associated with coding and noncoding sequences of cancer genomes [6]–[9]. These studies measured a higher proportion of base substitutions in MMR-deficient compared to MMR-proficient cancers [6]. Both MSI and large-scale mutational screenings only reveal mutations occurring in most cancer cells, namely in an expanded clonal population, while neglecting low-frequency substitutions. The returned picture is a “static snapshot” of the cancer genome in which only the tip of the iceberg (i.e., clonal mutations) is captured. The detection of low-frequency mutations in addition to clonal mutations is instrumental to clarify controversial aspects of cancer genetics. For example, the high sensitivity needed to find nonclonal mutations helps to trace the appearance of the mutator phenotype, thus clarifying the role of genomic instability during the early stages of carcinogenesis. So far, technical limitations prevented the detection of low-frequency mutations, since traditional sequencing procedures cannot reach the required level of sensitivity. In past years, several approaches have been explored to overcome this problem, often based on complex experimental settings [10],[11]. In principle, next-generation sequencing technologies could offer a valid solution, as they rely on amplification and sequencing of distinct DNA filaments. Because sensitivity of these methods increases with coverage, rare mutations should become detectable by performing an ultradeep resequencing of a given DNA region. The obvious drawback is connected with specificity: at deep coverage, low-frequency substitutions are an indistinguishable mixture of technical errors and true mutations, which makes it hard to distinguish true signal from noise. One possible solution to overcome technical errors is to use internal controls, i.e., genomic elements that do not accumulate true mutations so that all substitutions observed in these regions are bona fide errors. Ultraconserved regions (UCRs) of the human genome constitute a possible repository of such immutable segments. UCRs are genomic elements longer than 200 base pairs (bp), 100% identical between human, mouse, and rat, and significantly depleted in SNPs [12] and copy number variants [13] within the human population. Although mice lacking UCRs are in general viable and fertile [14], these regions undergo purifying selection [15] even stronger than nonsynonymous sites [16]. UCRs seem to have ideal features to be exploited as a control for the experimental errors of DNA amplification and sequencing. The working hypothesis is that by comparing the mutability of UCRs with that of genomically unstable regions, the higher mutation rate of the latter should become eventually detectable. This model works only under two assumptions. The first one is that UCRs are conserved, not only in germline, but also in somatic cells. Recently, an altered expression of some UCRs has been reported in leukaemia and carcinomas [17], and two out of six SNPs that are present in UCRs show significant association with familial breast cancer risk [18]. Both these studies suggest that UCRs may play a role also in adult cells, and therefore, they might be under somatic selection. The second assumption is that the cancer mutation rate is higher or at least comparable to the experimental error rate, because only in this case can the difference in mutability be appreciated. This seems a plausible assumption, given the current estimations for the cancer-associated mutator phenotype [10],[11].

As a proof of principle of this analytical approach, we resequenced more than 45,000 distinct DNA filaments of an ∼1,500-bp genomic segment centred on a carefully selected UCR. The region derived from three different tissues of patients affected by HNPCC: neoplastic colon mucosa, nonneoplastic colon mucosa, and peripheral blood. As a negative control, we used the peripheral blood of nine healthy donors. To amplify and sequence each sample, we used emulsion PCR followed by pyrosequencing [19]. This method offers, to date, the best compromise between sufficiently long reads and low error rate in miscalled bases [20]. The depth of coverage that we reached allowed us to detect genomic instability in neoplastic as well as in nonneoplastic HPNCC samples, offering the first, to our knowledge, evidence of constitutional genomic instability of these individuals.

## Results

### UCR Selection, Amplification, and Sequencing

**Figure 1:**
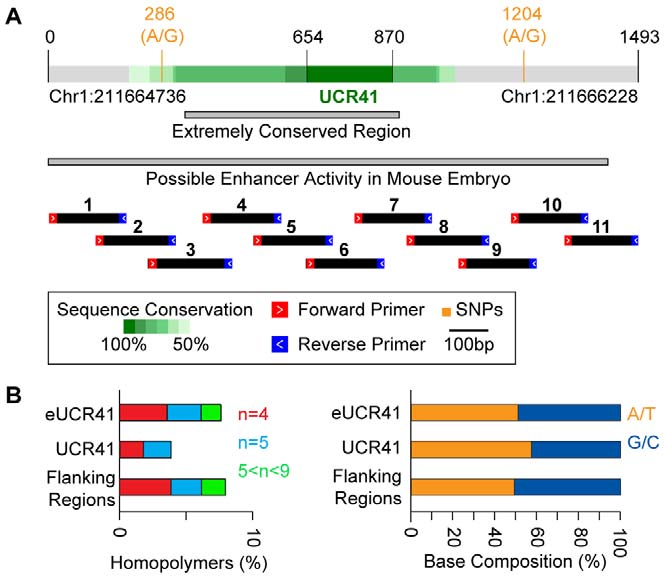
Features of eUCR41. (A) Genomic coordinates refer to the hg18 assembly of the human genome. The two grey bars correspond to the extremely conserved sequence [58], and to the genomic region tested for possible enhancer activity [21], respectively. Black bars indicate the 11 overlapping segments used for the amplification. (B) Percentage of homopolymers and base composition of eUCR41, of the ultraconserved core, and of the flanking regions are shown.

Starting from 481 UCRs [12], we restricted the analysis to the 307 regions detectable in seven fully sequenced vertebrates (human, mouse, rat, cow, chicken, frog, and fugu). We enlarged all UCRs in both directions to allow the inclusion of nonconserved sequences. The resulting extended UCRs (eUCRs) were composed of the ultraconserved core and nonconserved flanking regions. All 307 eUCRs were screened for genomic and functional properties that would favour the detection of a difference in mutability between the ultraconserved core and the flanking segments (Table S1). The best candidate was eUCR41, a 1,493-bp-long region centred on a 217-bp-long ultraconserved core (Figure 1A). This extended region bears two SNPs frequent in the European population, has no coding activity, and is located in a gene desert. Although the role of UCR41 is unknown, it has been reported to drive gene expression in the mouse embryo [21] and might be transcribed in adult cells [17]. We verified that homopolymers in eUCR41 are shorter than 10 bp and contribute for only a small portion of the entire region (∼8.2%). In addition, the base composition is similar inside and outside the ultraconserved core (Figure 1B).

We extracted the DNA from the neoplastic colon mucosa, nonneoplastic colon mucosa, and peripheral blood of nine HNPCC patients with known germline mutations in either *MLH1* or *MSH2* genes. All tumour samples, six adenocarcinomas, and three adenomas, were verified to display high degree of MSI (Table S2). As a negative control, we used the peripheral blood of nine healthy donors. To amplify eUCR41, we divided the region into 11 overlapping segments (Figure 1A) and reduced the PCR errors by using the highest fidelity DNA polymerase available to date [22]. To uniformly cover the region and minimize the contribution of single individuals, we pooled equimolar ratios of all amplicons from the different tissues types of each individual into four distinct samples: cancer colon (CC), nonneoplastic colon (NC), peripheral blood leukocytes (PBL), and healthy peripheral blood leukocytes (H-PBL). Each sample was sequenced on both sides using a fully dedicated run of ultradeep pyrosequencing [19]. This allowed sequencing of more than 83 million single bases per sample, corresponding to an average coverage of more than 45,000 reads/base pair (Figure S1, Table 1). After aligning all obtained reads to the reference sequence, we measured the substitution frequency at each position, defined as the percentage of reads bearing a nucleotide different from the reference. We distinguished between high (>0.1%) and low (<0.1%) frequency substitutions (Table 1), according to the estimated detection power of the method [23],[24].

**Table 1:** Results of the ultradeep sequencing screening.

Sample	Total Reads	Total Bases	Average Read Length (bp)	Aligned Reads	Positions with High Substitution Frequency (>0.1%)			Positions with Low Substitution Frequency (<0.1%)
					SNPs	Clonal Mutations	Errors	
CC	460,584	89,958,949	195.3	99.8%	2	2	20	1,221
NC	429,940	83,376,393	193.9	98.9%	2	0	18	1,215
PBL	496,358	96,210,962	193.8	99.8%	2	0	35	1,151
H-PBL	459,691	88,625,322	192.8	99.4%	2	0	38	1,157

For each sample, the total number of sequence reads and sequenced bases are shown, together with the average length of the reads and the percentage of reads aligned to the reference sequence. The latter correspond to the fraction of reads that passed the quality filter of 454 sequencing. Reported also are the positions of eUCR41 with substitutions at high (>0.1%) and low (<0.1%) frequency. The threshold of 0.1% represents the detection power of 454 sequencing.

### Analysis of High-Frequency Substitutions

After manual inspection, we discarded all but four high-frequency substitutions (Table 1). Errors were mostly generated by incorrect indels in proximity of polynucleotide stretches, often at the end of the reads where the sequencing performance decreases (Table S3). Indels caused misalignments between the reads and the reference sequence, which resulted in false substitutions (Figure S2).

**Figure 2:**
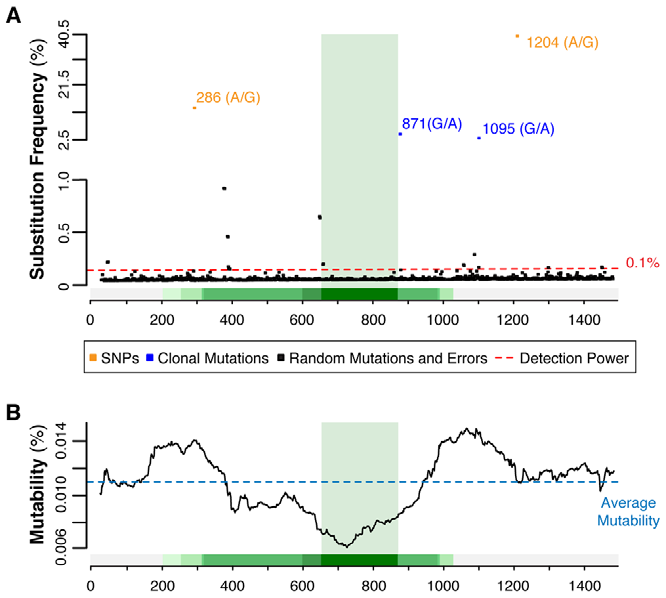
Mutation spectrum of eUCR41 in sample CC. (A) All detected substitutions are mapped on the corresponding positions of eUCR41. Two ranges of substitution frequency are shown: 40.5%–2.5% and <1.0%, since no substitution was detected in the range 2.5%–1.0%. All substitutions reported in the range 1.0%–0.1% were manually checked and excluded as sequencing errors. (B) Mutability was calculated using sliding windows of the same length as UCR41. Values corresponding to the middle point of each window are reported. Mutability increases with the decrease of sequence conservation: it is always below average for sequence identity >50%, whereas it is above average for nonconserved segments. Similar trends were observed for all samples deriving from HNPCC (unpublished data).

Of the four high-frequency mutations that passed the manual inspection, two are the known SNPs detectable in all four samples and two are G∶C to A∶T clonal somatic transitions only present in sample CC (Figure 2A). We genotyped eUCR41 in all analyzed individuals (Table S4) and confirmed that the minor allele frequency (MAF) of the two SNPs obtained with 454 sequencing was comparable with that inferred from Sanger sequencing (Table 2). This confirms that amplicons from the nine individuals were pooled in equimolar ratios in all four samples and that all of them contributed uniformly to the results. Sanger sequencing also showed that the two somatic mutations are detectable in heterozygosis in two different patients (patients 5 and 6, Table S4). From the substitution frequency obtained from pyrosequencing (Table 2), we could infer that mutations 871 and 1,095 occur in 37.0% and 23.4% of the corresponding PCR products, respectively. Considering that both are heterozygous, these mutations are present in about 74% and 47% of the diploid cancer genomes of patients 6 and 5, respectively. They therefore reflect the expansion of the dominant neoplastic clones. Further experimental validations are needed to assess whether these two clonal mutations are driver or passenger. The fact that both correspond to the wild-type nucleotide in mouse (A∶T) suggests that they might be tolerated, and hence hitchhiked, during clonal expansion.

**Table 2:** MAF of the high-frequency mutations in eUCR41.

Mutation	MAF in HNPCC Patients				MAF in Healthy Donors	
	Sample CC (454)	Sample NC (454)	Sample PBL (454)	Sanger	Sample H-PBL (454)	Sanger
SNP 286 (A/G)	13.7%	10.6%	12.0%	11.1%	4.5%	5.5%
SNP 1204 (A/G)	40.0%	42.0%	38.0%	38.9%	32.6%	33.3%
MUT 871 (G/A)	4.1%	—	—	—	—	—
MUT 1095 (G/A)	2.6%	—	—	—	—	—

For both SNPs and somatic mutations (MUT), the MAF in all samples is reported, as derived from 454 and Sanger sequencing. In the case of 454, MAF was calculated as the percentage of reads bearing the minor allele in each sample. In the Sanger screening, it corresponds to the fraction of minor alleles detected in the nine patients and in the nine healthy donors. Sanger genotyping confirmed that the two clonal mutations in sample CC are heterozygous mutations present in two different patients. Combining this information with the frequency in the 454 screening, it is possible to infer that these mutations are present in about 74% and 47% of the cells of the two patients, respectively.

Because indels at homopolymers are a major source of sequencing errors in the 454 platform ([20] and Figure S2), we ignored this type of modification in our analysis. Despite the high rate of indels in all four samples, the only two 9-bp-long polyAs of eUCR41 are significantly more instable in the HNPCC samples than in the healthy control (Table S5).

### Instability of HNPCC Neoplastic and Nonneoplastic Genome

**Figure 3:**
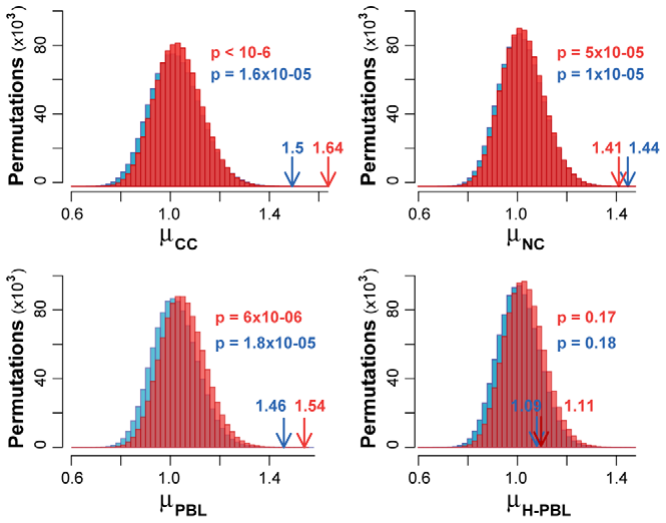
Observed and expected mutability outside and inside UCR41. Observed values of mutability ratios (arrows) were compared to the expected distributions computed from 1,000,000 random permutations of the raw data (red) and after removing all potential errors (blue). *p* represents the probability of obtaining the observed mutability ratio by chance and was calculated as the fraction of the expected ratios equal or higher than the observed value.

Low-frequency substitutions (<0.1%) likely consist of an indistinguishable mixture of nonclonal true mutations and errors that have been introduced during DNA amplification and pyrosequencing. Similarly to what we did for high-frequency substitutions, we excluded indels from the analysis to reduce the impact of 454 sequencing errors. The pattern of these substitutions is different, and their frequency is lower (Table S6) than the recently estimated contribution of PCR errors [25]. This is likely due to the fact that we used the polymerase with the lowest error rate compared to all other thermostable polymerases with 3′-5′ proofreading activity [22],[26],[27]. We used all low-frequency substitutions to measure the mutability of eUCR41, defined as the substitution frequency over the entire region (see Materials and Methods). To verify whether UCR41 is conserved also in cancer cells, we dynamically scanned the mutability within eUCR41 using sliding windows as long as UCR41. Whereas nonconserved segments of eUCR41 always show mutability higher than average, mutability decreases for increasing values of sequence conservation and reaches the minimum in correspondence of the ultraconserved core (Figure 2B). To assess the significance of the inverse correlation between mutability and sequence conservation, we compared the distribution of substitution frequency within the ultraconserved core with that of the flanking regions. We found that the two distributions differ significantly in neoplastic and nonneoplastic HNPCC samples, but not in healthy donors (Table 3). To exclude a possible bias due to the differences in length and, although minimal (Figure 1B), in base composition between UCR41 and its flanking segments, we measured the mutability ratio (μ) between flanking regions and UCR41 in all four samples. Each observed value was then compared to the expected distribution of mutability ratios after 1,000,000 random permutations. This comparison showed that base substitutions occur significantly more frequently in the flanking regions than in the ultraconserved core in all HNPCC samples but not in healthy donors (Figure 3).

**Table 3:** Substitution frequency and mutability outside and inside UCR41.

Sample	All Data					Data after Removing Potential Errors				
	Positions with Low Substitution Frequency		Median Substitution Frequency (×10^−3^)		*p*-Value	Positions with Low Substitution Frequency		Median Substitution Frequency (×10^−3^)		*p*-Value
	Outside	Inside	Outside	Inside		Outside	Inside	Outside	Inside	
CC	1,044	177	7.98	5.85	3×10^−7^ a	729	115	9.11	7.23	7×10^−4^ a
					2×10^−7^ b					4×10^−4^ b
NC	1,038	177	8.38	7.14	6×10^−5^ a	704	131	9.21	7.24	6×10^−5^ a
					3×10^−5^ b					3×10^−5^ b
PBL	979	172	7.82	5.28	8×10^−7^ a	655	114	8.73	6.95	5×10^−5^ a
					4×10^−7^ b					3×10^−5^ b
H-PBL	985	172	10.35	8.14	0.09^a^	672	111	10.85	10.34	0.99^a^
					0.05^b^					0.50^b^

For each sample, the number of positions with low substitution frequency and the median substitution frequency outside and inside UCR41 are reported, considering all data and after removing potential errors. At such a low substitution frequency, it is not possible to directly compare substitution frequencies between different samples because of the high contribution of run-specific errors. When the distributions of substitution frequency outside and inside UCR41 are compared in each sample, it becomes clear that they differ significantly in all three HNPCC samples, but not in H-PBL.

a Two-tailed Wilcoxon test.

b One-tailed Wilcoxon test (alpha value = 0.05).

### Control for Possible Amplification and Sequencing Errors

Because we rely on low-frequency substitutions for estimating genomic instability, it is instrumental to control for possible sources of noise that could invalidate our results. We therefore reanalysed the data after filtering for typical errors of the 454 platform. First, we removed all stretches of homopolymers (*n*>3) and two flanking bases on both sides, which are known to accumulate pyrosequencing artefacts [25]. Second, we removed all reads hosting at least one uncalled base, since they are prone to errors [28]. Finally, we discarded all substitutions occurring only in one read, which bear most random errors [24]. After removing all potential errors, the difference in substitution frequency (Table 3), as well as in mutability (Figure 3) between outside and inside UCR41 remains significant in all HNPCC samples and not significant in H-PBL. The same holds true when we applied the three filters separately (Table S7).

Although we used the highest fidelity polymerase, we further controlled whether PCR errors could have any impact on our results. We estimated that ∼12%–15% of low-frequency substitutions could be errors introduced by the DNA polymerase. After randomly removing a comparable fraction of substitutions in all four samples, we again observed higher mutability outside than inside UCR41 in HNPCC and no difference in H-PBL (Table S8). This test clearly excludes that PCR errors impacted in a significant manner on the observed difference in mutability between the UCR core and its flanking regions.

### Direct Comparison of HNPCC and Healthy Samples

**Figure 4:**
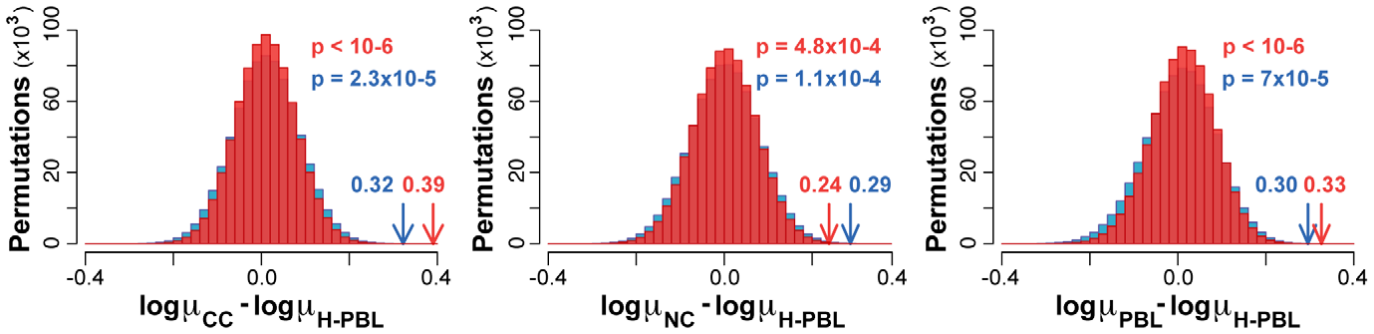
Difference in mutability ratio between HNPCC and healthy samples. The observed difference in mutability ratios (arrows) between each of the three HNPCC samples (μ_CC_, μ_NC_, and μ_PBL_) and the healthy control (μ_H-PBL_) were compared to the corresponding expected distributions. These were computed from 1,000,000 random permutations of the raw data (red) and after removing all potential errors (blue). *p* represents the probability of obtaining the observed difference in μ by chance and corresponds to the fraction of the expected differences equal or higher than the observed value.

Due to the occurrence of run-specific errors in the 454 platform [29], substitution frequencies of different samples cannot be compared directly. Instead of substitution frequencies, we compared the mutability ratios, which exploit the ultraconserved element to normalize the sample-specific errors. In particular, we compared the observed difference in mutability ratio between each of the HNPCC samples and H-PBL with the corresponding expected distribution. Also in this case, we performed 1,000,000 random permutations to compute expected differences in mutability ratios. In all three comparisons, the difference in the mutability ratio was significantly higher than expected using both raw and filtered data (Figure 4). This result provides further evidence that both neoplastic and nonneoplastic tissues from HNPCC patients accumulate more mutations than tissue from healthy individuals.

**Figure 5:**
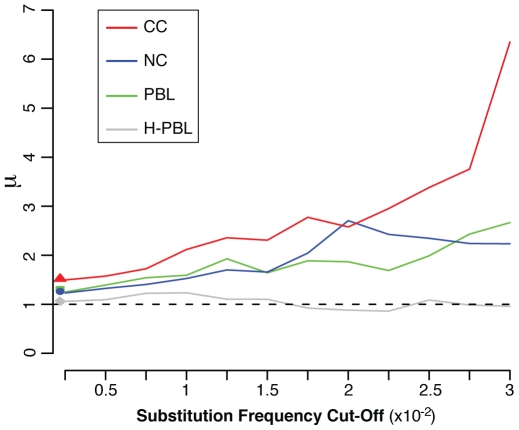
Variation of the mutability ratio for decreasing contribution of random errors. By progressively decreasing the number of positions with rare substitutions, the mutability ratio (μ) outside and inside UCR41 increases in all samples from HNPCC patients. In H-PBL, errors overcome true mutations inside and outside UCR41 at any value of frequency cutoff. The corresponding mutability ratio is therefore always around 1. Values on the *y*-axis correspond to the observed ratio for each sample.

Altogether, our data verify our initial assumption that UCR41 is maintained ultraconserved also in somatic cells, and it can be therefore used to normalize the experimental errors. At deep coverage, the mutation rate of the HNPCC genome allows detection of an increased occurrence of mutations in the flanking segments when compared to the ultraconserved core. No increase is detectable in the sample H-PBL, although UCR41 is very likely also to be conserved there. In this case, the mutation rate of the healthy human genome is so low that sequencing errors overcome true mutations in the entire region. The different behaviour between HNPCC and healthy samples becomes more evident when the contribution of random errors decreases. When we removed positions with substitutions at increasing values of frequency, the mutability ratio increases in all HNPCC samples, but not in H-PBL, where it is always around 1 (Figure 5). This result also excludes that the mutability ratio of the normal sample is due to a casual and nonhomogenous distribution of low-frequency substitutions between the ultraconserved core and the flanking segments.

### Sensitivity and Specificity in Detecting Rare Substitutions

In order to experimentally assess the error rate associated with pyrosequencing, we performed a controlled dilution experiment in which an amplicon carrying a single mutation (G, corresponding to the SNP at position 1,204, Figure 1A, Table S4) was diluted with the corresponding wild-type amplicon (A). At each step of the four controlled dilutions (1∶1,000; 1∶2,000; 1∶5,000; and 1∶10,000), wild-type and mutant amplicons were first quantified separately to control for experimental inaccuracy and then pooled. The four samples were sequenced using four distinct lanes. Although the expected coverage was 70,000 reads/lane, we obtained around double the amount of reads for each sample, which indicates an optimal experimental setting (Table 4). By plotting the observed frequency of the mutated allele against the corresponding dilution, we observed a strict linear correlation (*R* ^2^>0.99) also for the most extreme dilution (Figure S3 and Table 4). This result assesses the high sensitivity of our procedure in detecting very rare mutations. The dilution experiment also allows an estimation of specificity, defined as the fraction of correct positions over the total sequenced positions. In the sequenced region, specificity starts to decrease for substitution frequencies lower than 0.05% (Table 4). Since specificity depends on the sequence composition and complexity, it is reasonable to think that the lower bound of specificity is different for longer and more complex regions. This supports the mandatory usage of an internal normalization of the experimental error, when substitutions at very low frequency are considered. Interestingly, the few positions with substitution frequency between 0.1% and 0.05% (less than 18 in all four samples) show an overall frequency higher in sample CC than in sample H-PBL, also without using UCR41 as an internal control (*p*-value = 8×10^−3^, Wilcoxon text). This again confirms that the signal improves by removing random errors (Figure 5).

**Table 4:** Sensitivity and specificity in detection of rare mutations.

Dilution	Total Reads	Mutated Reads (G)	Observed Frequency (%G)	Expected Frequency (%G)	Positions with Errors	Specificity
1∶1,000	151,118	110	0.073	0.1	0	1.00
1∶2,000	148,990	56	0.038	0.05	0	1.00
1∶5,000	144,307	30	0.021	0.02	6	0.96
1∶10,000	161,921	19	0.012	0.01	24	0.85

For each dilution value, the total number of sequenced reads, the number of reads bearing the mutated allele (G), and the observed and expected substitution frequency are reported. We considered errors all positions showing a substitution frequency equal to or higher than the corresponding frequency of the mutated allele. This allowed measuring of the specificity, defined as the number of true negatives (156−errors) over all variable positions (156).

## Discussion

We exploited the frozen status of UCR41 to increase sensitivity and specificity of ultradeep sequencing and hence quantify cancer-associated genomic instability. The obtained results offered several insights into cancer genetics. We provided the first indication that an ultraconserved element does not accumulate mutations in somatic cells also in conditions of genomic instability. This result suggests that genomic instability is not constant in all regions of the cancer genome and that certain genomic portions are utterly preserved from modifications even in advanced tumoural stages such as carcinoma. It remains to be verified whether all UCRs are under the same somatic conservation and which are the reasons for it. In the case of UCR41, the extreme conservation could be a sign of strong purifying selection. UCR41 seems to be involved in a variety of different functions. It drives the expression of reporter gene in mouse embryos, [21], and gets transcribed into noncoding RNAs in adult tissues [17]. In addition, UCR41 is located upstream to *PROX1*, a gene that acts as a tumour suppressor in breast and pancreatic cancers [30],[31], hepatocellular carcinomas [32] and lymphomas [33]. Recently, *PROX1* has been shown to promote tumour growth and malignant progression in colorectal cancers [34]. Finally, the region between UCR41 and *PROX1* can undergo genomic rearrangements that have been associated with heart defects [35]. Altogether, these observations may indeed indicate that UCR41 is under functional constraints in both germline and somatic cells, although the alternative hypothesis of UCR41 as a cold spot for mutations, as proposed for other UCRs [14], cannot be completely ruled out. Whatever the biological reason for the somatic conservation of UCR41 may be, we proved that it can be used as an internal control for the sequencing errors, thus increasing the sensitivity in the detection of genomic instability.

This increased sensitivity led to the observation that the genome of nonneoplastic HNPCC cells has a constitutional mutation rate higher than MMR proficient genomes and, therefore, it is deficient in repairing DNA (Figure 4). Despite sporadic reports of low-frequency MSI [36],[37], HNPCC nonneoplastic cells are commonly assumed to repair DNA normally [38],[39]. This was based on measures of genomic instability that required the presence of clonal mutations. These assays were able to detect instability in tumoural samples, but not in pretumoural stages in which cells do not have a clonal origin. Indicative of the difference between the two approaches is the observation that several thousands of different clones are needed to reproduce the data reported here, with the concrete possibility of cloning PCR errors. The constitutional instability of MMR^+/−^ genomes implies that they start accumulating low-frequency substitutions before cancer transformation. This constitutional instability could predispose MMR^+/−^ individuals to the inactivation of the second allele, which is a mandatory step to initiate carcinogenesis [38],[39]. Known mechanisms of somatic inactivation of the MMR wild-type allele include loss of heterozygosity (LOH), promoter hypermethylation and somatic mutations in the gene sequence. The relative contribution of these three main mechanisms is controversial. In general, LOH seems the most common, with a frequency that ranges from 33% to 86% of the cases [40]–[46]. Although more rarely, somatic inactivating mutations have also been reported [4],[42],[43],[46]–[50]. In addition, there are a number of cases in which none of the known inactivating mechanisms can explain MMR deficiency [46],[47]. A constitutional mutation rate higher than healthy genome could contribute to an explanation of those cases, because deleterious mutations could directly hit the gene sequence, as well as other regions important, for example, for the regulation of gene expression. Our findings highlight the importance of an early diagnosis of genomic instability for selecting the best clinical approach to monitor, prevent, and possibly slow down the progression to cancer. A molecular test to reveal cancer predisposition could also restrict invasive surveillance examinations, such as colonoscopy and/or extracolonic screening of endometrium and ovary, only to positive carriers. To date, predisposition testing in family members with the Lynch syndrome consists of genetic screening of the MMR genes to identify germline mutations [51],[52]. Our strategy constitutes the proof of principle to implement an alternative test for diagnosing cancer predisposition without any a priori knowledge of the mutated genes. Although promising, several aspects of our procedure need further investigation. It remains to be confirmed whether MMR^+/−^ genomes of healthy carriers, (i.e., gene carriers who had not developed cancer yet) are unstable as well. So far, we have only analyzed nonneoplastic cells of HNPCC patients, which constitutes reliable, but indirect, evidence that this could indeed be the case. In addition, although the MAF inferred with Sanger was comparable with that obtained with 454 sequencing (Table 2), we cannot exclude that the mutation rate is variable even between individuals and not only between HNPCC carriers and healthy donors. We therefore need to measure genomic instability of single individuals to check for possible interindividual variability, DNA quality, and other technical factors, as well as to confirm the suitability of our approach as a genetic marker.

## Materials and Methods

### Ethics Statement

All individuals involved in this study agreed to and signed the informal consent form for the use of their biological samples for research purposes, approved by the local ethical committee in accordance with current Italian regulations.

### UCR Selection

The genomic coordinates of 481 UCRs were derived from the hg18 release of the human genome (March 2006). The conservation between each human UCR and the corresponding orthologous element in mouse (February 2006), rat (November 2004), dog (May 2005), cow (March 2005), chicken (February 2004), and fugu (August 2002) was derived from the multiZ alignments [53]. Only 307 UCRs detectable in all seven species were retained for further analysis. These UCRs were extended on both sides up to 50% of sequence conservation, measured as the percentage of nucleotides over a 25-bp sliding window conserved in at least four of the seven species. To include also nonconserved segments, regions were further extended 500 bp on both sides. The selection of extended UCR41 (eUCR41) as the best candidate for ultradeep sequencing was done as reported in Table S1. The entire sequence of eUCR41 was divided into 11 overlapping segments (amplicons), each around 200-bp long. For each amplicon, a pair of forward and reverse primers was designed with 40%–60% of GC content and a melting temperature of 58–60°C. The UCSC in silico PCR tool was used to check that selected primers did not have spurious additional matches on the human genome. All primers were fused with ad-hoc 5′ overhangs to allow emulsion PCR and sequencing.

### Sample Preparation and Sequencing

Nine HNPCC carriers were selected from the Registry of Hereditary Colorectal Cancer at the Istituto Nazionale Tumori (Milan, Italy). Heterozygous *MLH1* and *MSH2* mutations were detected on genomic DNA purified from peripheral blood leukocytes [54]. Nine healthy controls more than 50 years old (four males and five females) were selected among blood donors with Italian ancestry and no personal history of cancer. Tumours (six adenocarcinomas and three adenomas) and normal colonic mucosa were surgical removed and cryoconserved. Hematoxylin-eosin staining revealed that tumour areas were not heavily contaminated with normal cells, did not present necrosis, and that normal colonic mucosa was free of tumour infiltration. Tumour and matched normal DNAs were amplified by PCR using fluorescent primers followed by gel electrophoresis on a 3130 DNA Sequencer (Applied Biosystems) and fragments were analyzed using GeneScan and Genotyper software [55]. All tumour samples used for the analysis showed altered electrophoretic pattern in tumour compared with normal DNA for at least two microsatellites of the National Cancer Institute–recommended panel [56]. Genomic DNA was extracted from frozen tumours and normal mucosa using the QIAmp DNA Mini Kit and from PBL using the QIAmp DNA Blood Mini Kit (Qiagen) according to the manufacturer's instructions. Genomic DNA was amplified by PCR using the high-fidelity Pwo SuperYield DNA Polymerase (Roche). The PCR products were individually checked on agarose gel and purified using the AGENCOURT AMPure kit (Beckman Coulter) according to the manufacturer's protocol. All 99 amplicons from each tissue type (CC, NC, PBL, and H-PBL) were quantified using NanoDrop ND-1000 UV-Vis Spectrophotometer and pooled in equimolar ratio to obtain four samples (CC, NC, PBL, and H-PBL). Four independent runs of pyrosequencing were performed at 454 Life Sciences, each of them on a 70×75-mm PicoTiterPlate using the GS FLX Sequencer. Emulsion PCR and sequencing were performed as previously described [19]. Each sequence read was base called [19], filtered by quality metrics, and aligned to the human reference sequence as previously described [23]. Sanger sequencing was performed to characterize the genotype of each individual in each tissue and to identify the carriers of the two mutations in cancer. Amplicons were generated using the Pwo SuperYield DNA polymerase (Roche) and sequenced in both directions on a 3130×l sequencer, Data Collection 3.0 (Applied Biosystems), using the dRhodamine chemistry under standard conditions.

### Measures of Substitution Frequency, Mutability, and Mutability Ratio

For each position of eUCR41, the number of reads bearing a nucleotide different from the reference sequence was counted. The substitution frequency at position *j* was defined as:
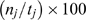
where *n* is the number of reads differing from the reference, and *t* is the total number of reads for position *j*. Positions with high substitution frequency (>0.1%) in all four samples were manually checked to reject possible false positives. In the analysis of positions with low substitution frequency (<0.1%), only base substitutions and no indels were considered to reduce the probability of pyrosequencing artefacts associated to insertions and deletions. Substitution frequency outside and inside UCR41 was compared using the Wilcoxon test.

The mutability of eUCR41 as well as of specific regions (i.e., ultraconserved core; flanking segments; 217-bp-long sliding windows) was defined as:
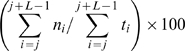
where *j* is the starting position and *L* is the length of the region. Mutability ratio (μ) was calculated as the ratio between mutability outside and inside UCR41:
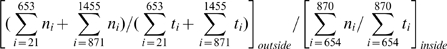



To account for the putative effects of length and base composition on the mutability of UCR41 and flanking segments, a permutation test was performed in which all positions with low-frequency substitutions were randomly reassigned in each sample, keeping the same length base composition of the two regions. Permutations were repeated 1,000,000 times, and the ratio between the expected mutability outside and inside UCR41 was calculated at each round. The probability (*p*) of observing the experimental ratio by chance was calculated as the fraction of the expected ratios equal or higher than the observed value.

The three null distributions to test the difference of mutability ratio between cases (samples CC, NC, and PBL) and control (sample H-PBL) were also computed using a permutation test. For each comparison, all sequence positions were randomly reassigned for 1,000,000 times, again maintaining length and base composition of UCR41 and flanking regions. At each permutation, the difference in the mutability ratio was derived, and each expected distribution was compared to the corresponding observed difference.

### Estimation of PCR Errors

The number of possible errors introduced by the DNA polymerase during the polymerase chain reaction (PCR errors), was first estimated and then removed from experimental data. PCR errors were quantified using two different approaches. The first one was based on the binomial probability distribution, in which the number of PCR errors *X* was considered a random variable that follows a binomial distribution:
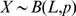
where *L* is the length of the region, and *p* is the probability to accumulate errors at a given position after *d* duplications with a given number of errors *r* introduced per base pairs at each duplication:
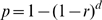



From this model, the total number of PCR errors expected in a region *L* is:
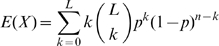



The total number N of PCR errors present in *n* single-stranded DNA sequences will be:
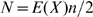



In our analysis, parameters *r*, *d*, *L*, and *n* were all derived from the experimental data. The applied error rate was *r* = 6.5×10^−7^ errors/base pair/duplication [22],[57]. The number of duplications was set equal to the number of PCR cycles *d* = 40. The length *L* of the region was calculated as the number of positions unchanged or bearing low-frequency substitutions in each sample (1,431; 1,435; 1,418; and 1,415 in CC, NC, PBL, and H-PBL, respectively). The number *n* of single-stranded DNA sequences was taken from the number of reads of each sample (49,194; 45,383; 53,212; and 49,005 in CC, NC, PBL, and H-PBL, respectively). In the second approach, the cycles of PCR amplifications were simulated in silico using a model similar to that used for the mutation rate. Starting from one DNA double strand of length *L*, errors were randomly introduced at a rate *r* in each position of the strand at each of the *d* PCR cycles. Once introduced, errors were retained in all the daughter strands. At the end of the amplification, the number of PCR errors present in the *n* single strands of DNA sequences was derived. The procedure was reiterated 1,000 times to generate a distribution of N values. The number of estimated PCR errors returned by the two approaches is identical and is reported in Table S8.

To verify the putative effect of PCR errors on the difference in mutability originally detected between the UCR core and the flanking regions, a number of low-frequency substitutions equal to the estimated number of PCR errors in each sample was randomly removed. The procedure was repeated 1,000 times, and the distribution of observed mutability ratios between the flanking regions and the UCR core was derived. Applying the same permutation used for the real samples, the distribution of expected ratios was also derived. The results of both simulations are reported in Table S8, together with the *p*-values of the comparison between observed and expected distributions.

All statistical analyses were performed using the R statistical environment and ad hoc Perl scripts.

### Serial Dilution

Dilution experiments were performed using the 157-bp-long segment of eUCR41 corresponding to amplicon 9, which bears a SNP in position 1,204 (SNP A/G, Figure 1A). This segment was amplified from the blood of two healthy donors showing homozygous AA and GG genotypes, respectively (Samples 13 and 14, Table S4). After amplification, the regions were purified as described above and pooled in different relative amounts. Four final dilutions were obtained with decreasing G∶A ratios (1∶1,000; 1∶2,000; 1∶5,000; and 1∶10,000; respectively). To correct for possible experimental inaccuracies during DNA quantification and pipetting, at each step of the serial dilutions, DNA quantifications of the two alleles were performed using the Victor PicoGreen fluorometer (PerkinElmer Life Sciences). The obtained values were used to calibrate the successive dilution. The DNA samples corresponding to the four dilutions were sequenced using four distinct lanes using a four-lane gasket for 70×75 PicoTiterPlate device on the GS FLX Sequencer at BMR Genomics. Specificity was measured as TN/(TN+*FP*). The number of true negatives (TN) was calculated as the number of correctly sequenced positions, i.e., positions with no errors at a frequency equal or higher than the frequency of the diluted allele.

## Supporting Information

Figure S1 **Depth of coverage reached with the sequencing screenings.** For each sample, the coverage of sequencing (reads/base pair) was measured. The average coverage is 49,150 in sample CC; 45,370 in sample NC; 52,530 in sample PBL; and 48,380 in sample H-PBL. Regions in which the coverage almost doubles correspond to overlapping segments between contiguous amplicons (see Materials and Methods and Figure 1A). Colour gradient corresponds to the degree of sequence conservation, as reported in Figure 1A. UCR41 is highlighted in green.
(6.29 MB TIF)

Figure S2 **Examples of high-frequency errors.** For each of the four hot spot regions described in Table S3, a different example of high-frequency errors derived from sample CC is shown. In all cases, the errors are due to indels that cause misalignments between the reads and the reference sequence. In three cases, the misaligned region corresponds to the end of the reads (*). (A) Reference position 1,050–1,061, frequency 0.1%. (B) Reference position 633–652, frequency 0.6%. (C) Reference position 1,071–1,094, frequency 0.1%. (D) Reference position 29–45 frequency 0.1%.
(0.62 MB TIF)

Figure S3 **Sensitivity In detecting rare mutations.** Serial dilution of amplicon 9 bearing a SNP in position 1,204 (G, Figure 1A) to the corresponding wild-type amplicon (A). The linear regression curve was calculated by plotting the observed frequency of the mutated allele G for a series of dilutions into the corresponding A wild-type allele. A strict linear correlation is maintained between observed and expected substitution frequency also for allele frequency of 0.01% (dilution 1∶10,000).
(0.13 MB TIF)

Table S1 **Criteria for the selection of eUCR41 for ultradeep sequencing.** Shown are the genomic and functional features, the reasons why they are important for the selection of the best eUCR, the detection methods, and the corresponding properties of eUCR41, the selected candidate. CEU, Utah residents with ancestry from northern and western Europe; dCNE, duplicated conserved noncoding elements.
(0.07 MB DOC)

Table S2 **HNPCC samples used for the analysis.** For each HNPCC patient, sex, germline mutation, histological properties, and level of microsatellite instability (MSI) are indicated. Germline mutations are described following the guidelines of the Human Genome Variation Society (http://www.hgvs.org/mutnomen). MSI was assessed in both adenomas and adenocarcinomas by checking for the presence of at least two unstable microsatellite markers (BAT25 and BAT26) [1].
(0.05 MB DOC)

Table S3 **Manual inspection of positions with high-frequency errors.** For each type of error, the possible source, the range of positions in the reference sequence, and resulting positions with errors in all four samples are reported. Most sequencing errors occur in close proximity of stretches of polynucleotides and result in hot spots of false insertions and deletions (indels). Indels also cause misalignments with the reference sequence, with consequent false substitutions. Representative flowgrams are shown in Figure S2 for all four main error hot spots. In four positions, the sequencing errors are due to miscalls. We considered them as false substitutions because either they had similar substitution frequency in all four samples (positions 116, 1,444, and 1,445), or they were present only in one sequencing direction (position 345, present only in reverse amplicons). In these cases, we do not show any flowgram because they are not explicative of the error type.
(0.05 MB DOC)

Table S4 **Genotyping and confirmation of high-frequency mutations.** For both HNPCC patients (1–9) and healthy donors (10–18), the corresponding genotype of SNPs and somatic mutations in eUCR41 is reported in each analysed individual, as detected by Sanger sequencing. The genotype was used to measure the minor allele frequency (MAF), defined as the frequency of the rare allele over the total. The similar values of the MAFs obtained with Sanger and with 454 sequencing allowed us to confirm that the samples used in this study were pooled in equimolar ratios (Table 2). Clonal somatic mutations in sample CC of patients 5 and 6 are reported in red, whereas the individuals used for the dilution series are shown in blue. Blood of patient 1 was not available for further analysis.
(0.10 MB DOC)

Table S5 **Rate of indels at homopolymers in the four samples.** The percentage of reads with indels of at least 1 bp in the homopolymeric tract is reported for the two 9-bp-long polyAs in each sample.
(0.04 MB DOC)

Table S6 **Frequency and pattern of low-frequency substitutions.** For each type of substitution, the frequency was calculated as the number of times that the substitution was observed divided by the number of times that that position was read.
(0.05 MB DOC)

Table S7 **Comparison of substitution frequency and mutability outside and inside UCR41 after filtering for sequencing errors.** Reported is the number of positions with low substitution frequency (<0.1%) outside and inside UCR41 for each sample, after three different filters for sequencing errors were applied. After each filtering, the usual statistical analyses were applied. In particular, the distributions of substitution frequency outside and inside UCR41 were compared using the Wilcoxon test, whereas the observed mutability ratio was compared to the expected distribution after 1,000,000 random permutations (see main text). *Two-tailed Wilcoxon test (alpha value = 0.05). **Probability of observing a mutability ratio equal or higher than the observed value, after 1,000,000 random permutations.
(0.06 MB DOC)

Table S8 **Estimation of PCR errors.** For each sample, the total number of estimated PCR errors was derived using the binomial probability distribution. Comparable numbers were obtained using the simulation model (see text). The corresponding percentage of PCR errors over the total low-frequency substitutions (<0.1%) was calculated for raw data and after filtering for potential sequencing errors. As expected, the percentage of PCR errors increases after filtering for sequencing errors, since the contribution of errors introduced by 454 sequencing decreases. For observed and expected distributions of mutability ratios, the mean, as well as 95% confidence interval (in brackets), are reported. In each sample, observed and expected distributions were compared using the Wilcoxon test.
(0.05 MB DOC)

## Abbreviations

CC: cancer colon mucosa
eUCR: extended ultraconserved region
H-PBL: healthy peripheral blood leukocytes
HNPCC: hereditary non-polyposis colorectal cancer
MAF: minor allele frequency
MMR: mismatch repair
MSI: microsatellite instability
NC: nonneoplastic colon mucosa
PBL: peripheral blood leukocytes
UCR: ultraconserved region
