# Supplementary figures and images for: Ultradeep Sequencing of a Human Ultraconserved Region Reveals Somatic and Constitutional Genomic Instability
Source: PLoS Biol. 2010 Jan 5;8(1):e1000275. doi: 10.1371/journal.pbio.1000275 (PMC2794366; doi:10.1371/journal.pbio.1000275)

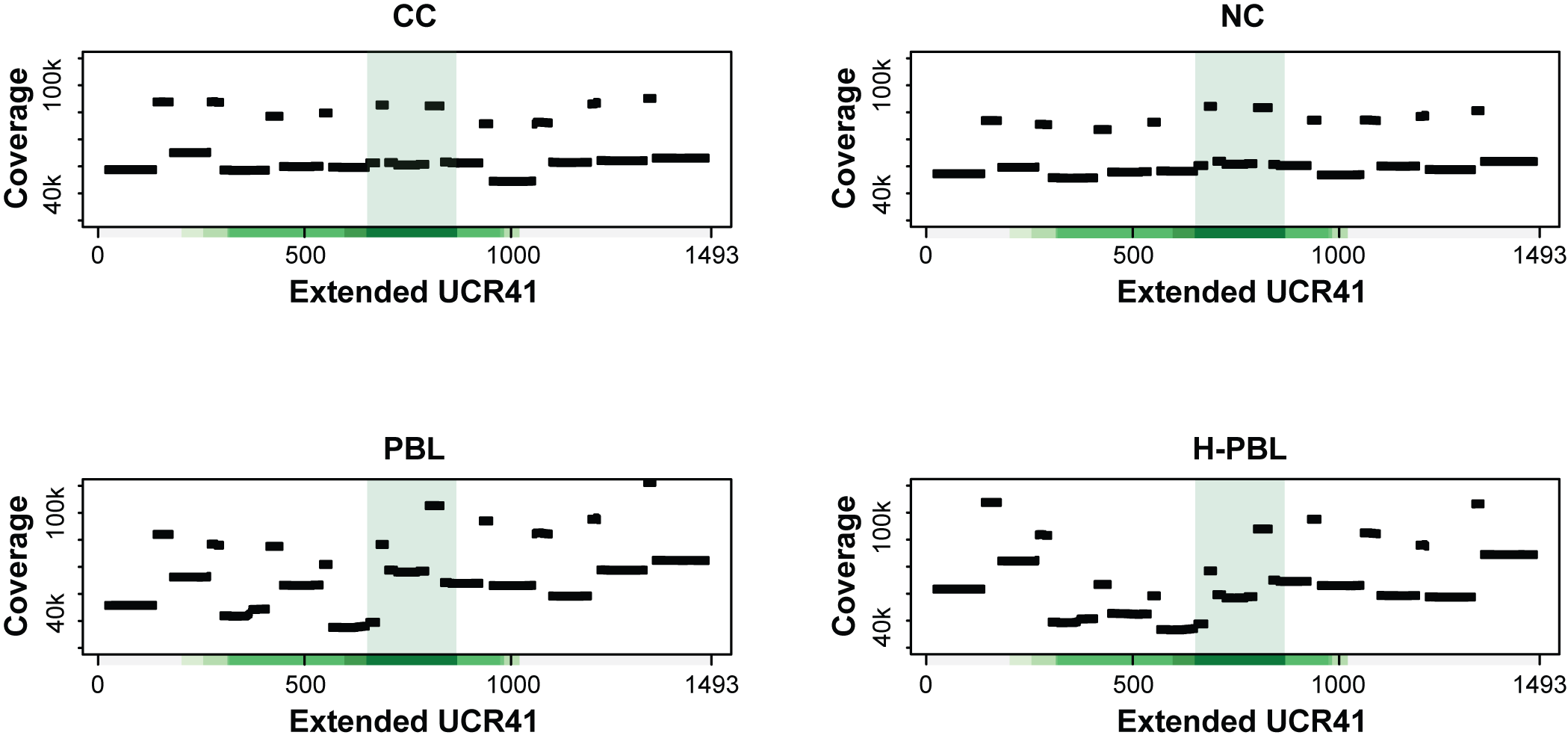

Supplement: Figure S1 — Depth of coverage reached with the sequencing screenings. For each sample, the coverage of sequencing (reads/base pair) was measured. The average coverage is 49,150 in sample CC; 45,370 in sample NC; 52,530 in sample PBL; and 48,380 in sample H-PBL. Regions in which the coverage almost doubles correspond to overlapping segments between contiguous amplicons (see Materials and Methods and Figure 1A). Colour gradient corresponds to the degree of sequence conservation, as reported in Figure 1A. UCR41 is highlighted in green. (6.29 MB TIF) [file pbio.1000275.s001.tif]

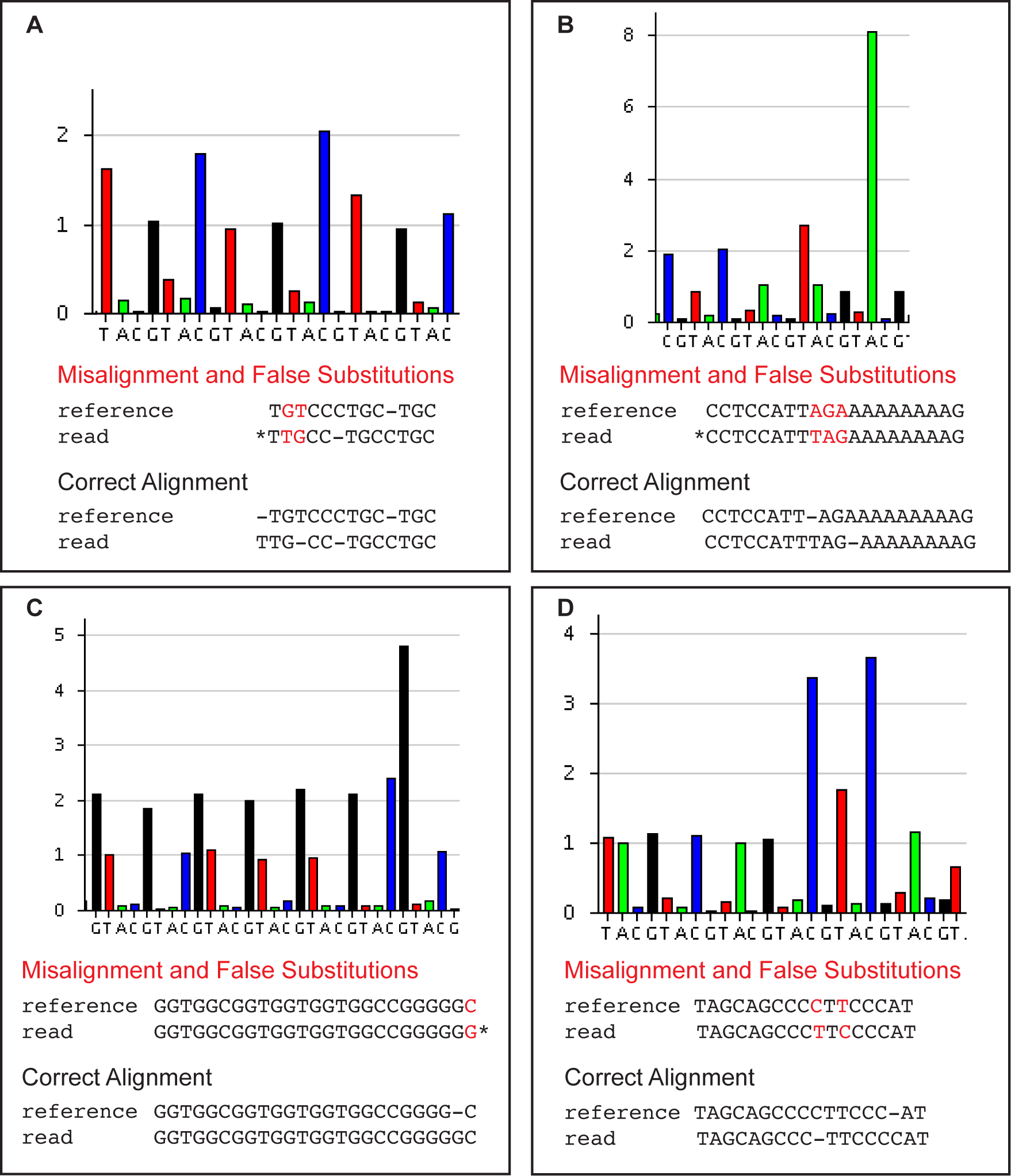

Supplement: Figure S2 — Examples of high-frequency errors. For each of the four hot spot regions described in Table S3, a different example of high-frequency errors derived from sample CC is shown. In all cases, the errors are due to indels that cause misalignments between the reads and the reference sequence. In three cases, the misaligned region corresponds to the end of the reads (*). (A) Reference position 1,050–1,061, frequency 0.1%. (B) Reference position 633–652, frequency 0.6%. (C) Reference position 1,071–1,094, frequency 0.1%. (D) Reference position 29–45 frequency 0.1%. (0.62 MB TIF) [file pbio.1000275.s002.tif]

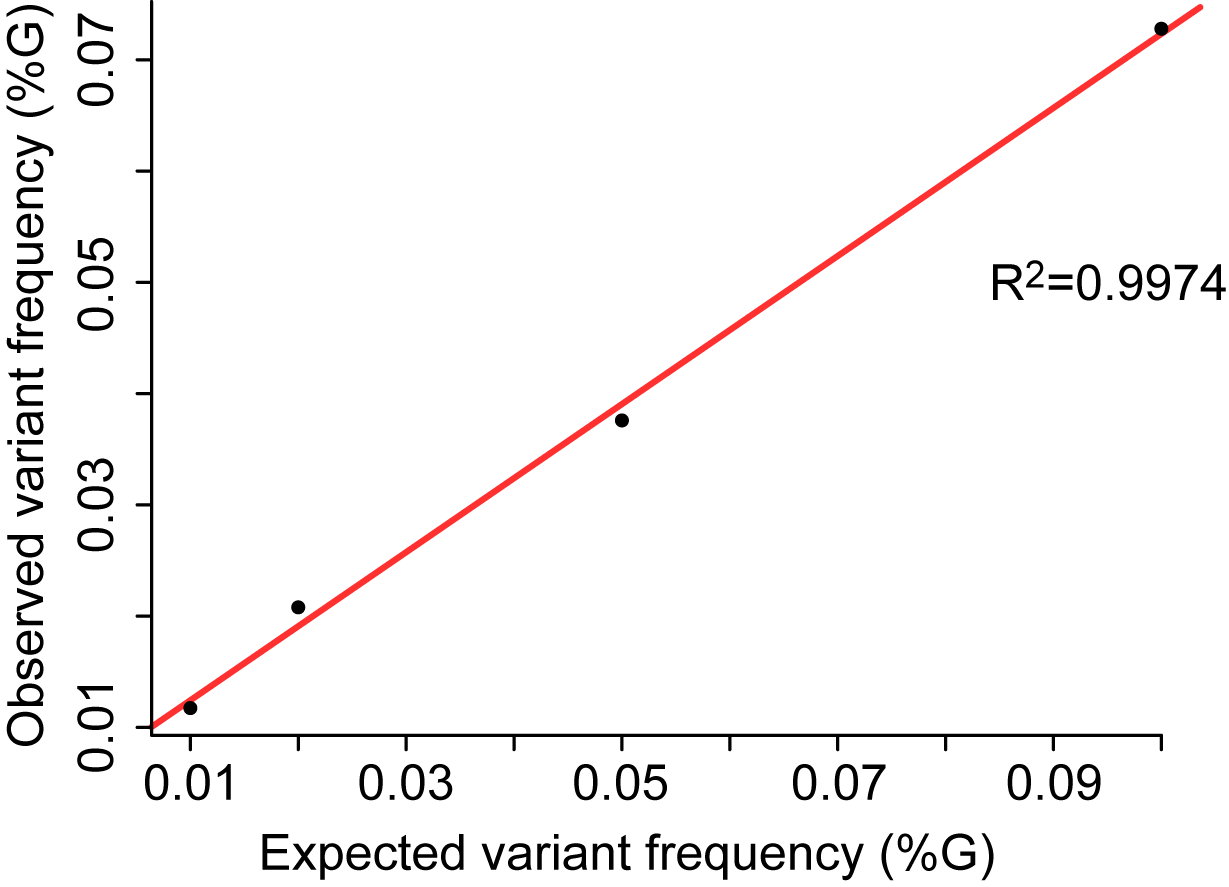

Supplement: Figure S3 — Sensitivity In detecting rare mutations. Serial dilution of amplicon 9 bearing a SNP in position 1,204 (G, Figure 1A) to the corresponding wild-type amplicon (A). The linear regression curve was calculated by plotting the observed frequency of the mutated allele G for a series of dilutions into the corresponding A wild-type allele. A strict linear correlation is maintained between observed and expected substitution frequency also for allele frequency of 0.01% (dilution 1∶10,000). (0.13 MB TIF) [file pbio.1000275.s003.tif]
