# Supplementary material for: Ultradeep Sequencing of a Human Ultraconserved Region Reveals Somatic and Constitutional Genomic Instability
Source: PLoS Biol. 2010 Jan 5;8(1):e1000275. doi: 10.1371/journal.pbio.1000275 (PMC2794366; doi:10.1371/journal.pbio.1000275)
Supplement: Table S1 — Criteria for the selection of eUCR41 for ultradeep sequencing. Shown are the genomic and functional features, the reasons why they are important for the selection of the best eUCR, the detection methods, and the corresponding properties of eUCR41, the selected candidate. CEU, Utah residents with ancestry from northern and western Europe; dCNE, duplicated conserved noncoding elements. (0.07 MB DOC) [file pbio.1000275.s004.doc]

**Table S1:** Criteria for the Selection of eUCR41 for Ultra-Deep Sequencing

| **Feature** | **Motivation(s)** | **Methods** | **eUCR41** |
| --- | --- | --- | --- |
| Flanking sequences with a sharp decay of sequence conservation | To compare mutability inside and outside the UCR | - MultiZ [1]  - PhastCons [2] | - UCR41 = 217 bp  - Flanking segments = 1238 bp |
| Absence of:  (1) simple repeats;  (2) duplicated regions;  (3) dCNEs | To exclude multiple mapping of primers and reduce sequencing errors | - Segmental duplications [3]  - RepeatMasker [4]  - dCNE [5] | Absence of duplications |
| No overlap with other UCRs | To unambiguously define boundaries of each UCR | Intersection of genomic coordinates | Unique UCR in the extended region |
| No coding activity and distance from the nearest gene >100 Kb | To exclude selective pressure directly associated to protein coding activity | Mapping of known genes, mRNAs, and ESTs | - No coding activity  - 373 Kb distant from *RPSKC1* and 563 Kb from *PROX1* |
| Functional evidence | To exclude that the region is a cold spot for mutations | *In vivo* assay for enhancer activity [6, 7] | Possible enhancer activity in the forebrain of mouse embryos and transcription on ncRNAs |
| Presence of SNPs common in the CEU population | To be used as internal control of high frequency mutations | - dbSNP [8]  - HapMap [9] | - rs17701179 (15% in CEU)  - rs3910657 (49% in CEU) |

**References**

1. Blanchette M, Kent WJ, Riemer C, Elnitski L, Smit AF, et al. (2004) Aligning multiple genomic sequences with the threaded blockset aligner. Genome Res 14: 708-715.

2. Siepel A, Bejerano G, Pedersen JS, Hinrichs AS, Hou M, et al. (2005) Evolutionarily conserved elements in vertebrate, insect, worm, and yeast genomes. Genome Res 15: 1034-1050.

3. Bailey JA, Yavor AM, Massa HF, Trask BJ, Eichler EE (2001) Segmental duplications: organization and impact within the current human genome project assembly. Genome Res 11: 1005-1017.

4. Jurka J (2000) Repbase update: a database and an electronic journal of repetitive elements. Trends Genet 16: 418-420.

5. McEwen GK, Woolfe A, Goode D, Vavouri T, Callaway H, et al. (2006) Ancient duplicated conserved noncoding elements in vertebrates: a genomic and functional analysis. Genome Res 16: 451-465.

6. Pennacchio LA, Ahituv N, Moses AM, Prabhakar S, Nobrega MA, et al. (2006) In vivo enhancer analysis of human conserved non-coding sequences. Nature 444: 499-502.

7. Calin GA, Liu C-g, Ferracin M, Hyslop T, Spizzo R, et al. (2007) Ultraconserved Regions Encoding ncRNAs Are Altered in Human Leukemias and Carcinomas. Cancer Cell 12: 215-229.

8. Sherry ST, Ward MH, Kholodov M, Baker J, Phan L, et al. (2001) dbSNP: the NCBI database of genetic variation. Nucleic Acids Res 29: 308-311.

9. Thorisson GA, Smith AV, Krishnan L, Stein LD (2005) The International HapMap Project Web site. Genome Res 15: 1592-1593.
