# Supplementary material for: Ultradeep Sequencing of a Human Ultraconserved Region Reveals Somatic and Constitutional Genomic Instability
Source: PLoS Biol. 2010 Jan 5;8(1):e1000275. doi: 10.1371/journal.pbio.1000275 (PMC2794366; doi:10.1371/journal.pbio.1000275)
Supplement: Table S2 — HNPCC samples used for the analysis. For each HNPCC patient, sex, germline mutation, histological properties, and level of microsatellite instability (MSI) are indicated. Germline mutations are described following the guidelines of the Human Genome Variation Society (http://www.hgvs.org/mutnomen). MSI was assessed in both adenomas and adenocarcinomas by checking for the presence of at least two unstable microsatellite markers (BAT25 and BAT26) [1]. (0.05 MB DOC) [file pbio.1000275.s005.doc]

**Table S2:** HNPCC Samples Used for the Analysis

| **Patient** | **Sex** | **Germline Mutation** | **Tumor Histology** | **MSI** |
| --- | --- | --- | --- | --- |
| 1 | M | MLH1: c.1852_1854delAAG (p.Lys618del) | Adenocarcinoma | High |
| 2 | F | MLH1: c.544+3A>G (p.Glu153fs) | Adenocarcinoma | High |
| 3 | M | MLH1: c.1852_1854delAAG (p.Lys618del) | Adenocarcinoma | High |
| 4 | M | MSH2: c.208-?_307+?del (p.Ala123_Gln215del) | Adenocarcinoma | High |
| 5 | M | MSH2: c.2294delC (p.Ala765fs) | Adenoma | High |
| 6 | M | MLH1: c.454+1G>A (p.Ala128fs) | Adenoma | High |
| 7 | M | MLH1: c.1050delA (p.Gly351fs) | Adenocarcinoma | High |
| 8 | F | MSH2: c.678-?_791+?del (p.Val463fs) | Adenocarcinoma | High |
| 9 | F | MSH2: c.2519_2530del12 (p.Val840_Gly843del) | Adenoma | High |

**Reference**

1. Boland CR, Thibodeau SN, Hamilton SR, Sidransky D, Eshleman JR, et al. (1998) A National Cancer Institute Workshop on Microsatellite Instability for cancer detection and familial predisposition: development of international criteria for the determination of microsatellite instability in colorectal cancer. Cancer Res 58: 5248-5257.
