# Supplementary material for: Ultradeep Sequencing of a Human Ultraconserved Region Reveals Somatic and Constitutional Genomic Instability
Source: PLoS Biol. 2010 Jan 5;8(1):e1000275. doi: 10.1371/journal.pbio.1000275 (PMC2794366; doi:10.1371/journal.pbio.1000275)
Supplement: Table S3 — Manual inspection of positions with high-frequency errors. For each type of error, the possible source, the range of positions in the reference sequence, and resulting positions with errors in all four samples are reported. Most sequencing errors occur in close proximity of stretches of polynucleotides and result in hot spots of false insertions and deletions (indels). Indels also cause misalignments with the reference sequence, with consequent false substitutions. Representative flowgrams are shown in Figure S2 for all four main error hot spots. In four positions, the sequencing errors are due to miscalls. We considered them as false substitutions because either they had similar substitution frequency in all four samples (positions 116, 1,444, and 1,445), or they were present only in one sequencing direction (position 345, present only in reverse amplicons). In these cases, we do not show any flowgram because they are not explicative of the error type. (0.05 MB DOC) [file pbio.1000275.s006.doc]

**Table S3:** Manual Inspection of Positions with High Frequency Errors

| **Error Type** | **Error Source** | **Position in the Reference** | **Number of Positions with High Frequency Errors** | | | | **Panel in Figure S2** |
| --- | --- | --- | --- | --- | --- | --- | --- |
|  |  |  | **CC** | **NC** | **PBL** | **H-PBL** |  |
| Indel and Misalignment | Trinucleotide Repeats | 1050-1070;  1077-1085 | 3 | 6 | 1 | 3 | A |
|  | PolyA | 363-386;  627-652 | 11 | 8 | 33 | 32 | B |
|  | PolyG | 1090-1094 | 1 | 0 | 0 | 0 | C |
|  | Adjacent polyNs | 35-43;  1287-1300 | 3 | 2 | 0 | 1 | D |
| Miscall | Sequencing Error | 116; 345; 1444; 1445 | 2 | 2 | 1 | 2 | NA |
