# Supplementary material for: Ultradeep Sequencing of a Human Ultraconserved Region Reveals Somatic and Constitutional Genomic Instability
Source: PLoS Biol. 2010 Jan 5;8(1):e1000275. doi: 10.1371/journal.pbio.1000275 (PMC2794366; doi:10.1371/journal.pbio.1000275)
Supplement: Table S4 — Genotyping and confirmation of high-frequency mutations. For both HNPCC patients (1–9) and healthy donors (10–18), the corresponding genotype of SNPs and somatic mutations in eUCR41 is reported in each analysed individual, as detected by Sanger sequencing. The genotype was used to measure the minor allele frequency (MAF), defined as the frequency of the rare allele over the total. The similar values of the MAFs obtained with Sanger and with 454 sequencing allowed us to confirm that the samples used in this study were pooled in equimolar ratios (Table 2). Clonal somatic mutations in sample CC of patients 5 and 6 are reported in red, whereas the individuals used for the dilution series are shown in blue. Blood of patient 1 was not available for further analysis. (0.10 MB DOC) [file pbio.1000275.s007.doc]

**Table S4:** Genotyping and Confirmation of High-Frequency Mutations

| **Sample** | **Individuals** | | | | | | | | |
| --- | --- | --- | --- | --- | --- | --- | --- | --- | --- |
| **CC** | **1** | **2** | **3** | **4** | **5** | **6** | **7** | **8** | **9** |
| SNP 286 (A/G) | AA | AA | AA | AA | AA | AG | AA | AA | AG |
| SNP 1204 (A/G) | AA | AG | AG | AA | AG | AG | AA | GG | AG |
| MUT 871 (G/A) | GG | GG | GG | GG | GG | GA | GG | GG | GG |
| MUT 1095 (G/A) | GG | GG | GG | GG | GA | GG | GG | GG | GG |
| **NC** | **1** | **2** | **3** | **4** | **5** | **6** | **7** | **8** | **9** |
| SNP 286 (A/G) | AA | AA | AA | AA | AA | AG | AA | AA | AG |
| SNP 1204 (A/G) | AA | AG | AG | AA | AG | AG | AA | GG | AG |
| MUT 871 (G/A) | GG | GG | GG | GG | GG | GG | GG | GG | GG |
| MUT 1095 (G/A) | GG | GG | GG | GG | GG | GG | GG | GG | GG |
| **PBL** | **1** | **2** | **3** | **4** | **5** | **6** | **7** | **8** | **9** |
| SNP 286 (A/G) | - | AA | AA | AA | AA | AG | AA | AA | AG |
| SNP 1204 (A/G) | - | AG | AG | AA | AG | AG | AA | GG | AG |
| MUT 871 (G/A) | - | GG | GG | GG | GG | GG | GG | GG | GG |
| MUT 1095 (G/A) | - | GG | GG | GG | GG | GG | GG | GG | GG |
| **H-PBL** | **10** | **11** | **12** | **13** | **14** | **15** | **16** | **17** | **18** |
| SNP 286 (A/G) | AA | AA | AA | AA | AA | AA | AA | AG | AA |
| SNP 1204 (A/G) | AG | AG | AG | AA | GG | AA | AA | AA | AG |
| MUT 871 (G/A) | GG | GG | GG | GG | GG | GG | GG | GG | GG |
| MUT 1095 (G/A) | GG | GG | GG | GG | GG | GG | GG | GG | GG |
