# Supplementary material for: Ultradeep Sequencing of a Human Ultraconserved Region Reveals Somatic and Constitutional Genomic Instability
Source: PLoS Biol. 2010 Jan 5;8(1):e1000275. doi: 10.1371/journal.pbio.1000275 (PMC2794366; doi:10.1371/journal.pbio.1000275)
Supplement: Table S5 — Rate of indels at homopolymers in the four samples. The percentage of reads with indels of at least 1 bp in the homopolymeric tract is reported for the two 9-bp-long polyAs in each sample. (0.04 MB DOC) [file pbio.1000275.s008.doc]

**Table S5:** Rate of Indels at Homopolymes in the Four Samples

| **Sample** | **% of Reads with ≥ 1 Indel** | |
| --- | --- | --- |
|  | **polyA (371-380)** | **polyA (642-651)** |
| CC | 68 | 68 |
| NC | 66 | 64 |
| PBL | 71 | 41 |
| H-PBL | 44 | 21 |
