# Supplementary material for: Ultradeep Sequencing of a Human Ultraconserved Region Reveals Somatic and Constitutional Genomic Instability
Source: PLoS Biol. 2010 Jan 5;8(1):e1000275. doi: 10.1371/journal.pbio.1000275 (PMC2794366; doi:10.1371/journal.pbio.1000275)
Supplement: Table S6 — Frequency and pattern of low-frequency substitutions. For each type of substitution, the frequency was calculated as the number of times that the substitution was observed divided by the number of times that that position was read. (0.05 MB DOC) [file pbio.1000275.s009.doc]

**Table S6:** Frequency and Pattern of Low Frequency Substitutions

| **Substitution Type** | **Sample**  **CC** | **Sample**  **NC** | **Sample**  **PBL** | **Sample**  **H-PBL** |
| --- | --- | --- | --- | --- |
| C:G->T:A | 9x10^-5^ | 8.9x10^-5^ | 8.2x10^-5^ | 9.2x10^-5^ |
| T:A->C:G | 4x10^-5^ | 4.31x10^-5^ | 3x10^-5^ | 4x10^-5^ |
| C:G->A:T | 2.1x10^-5^ | 2.5x10^-5^ | 3x10^-5^ | 4x10^-5^ |
| T:A->A:T | 1.9x10^-5^ | 1.7x10^-5^ | 1.8x10^-5^ | 1.8x10^-5^ |
| C:G->G:C | 1.1x10^-5^ | 8x10^-6^ | 5x10^-6^ | 6x10^-6^ |
| T:A->G:C | 1x10^-5^ | 9x10^-6^ | 9x10^-6^ | 1.2x10^-5^ |
| **Total** | **9.6x10^-5^** | **9.6x10^-5^** | **8.5x10^-5^** | **10.6x10^-5^** |
