# Supplementary material for: Ultradeep Sequencing of a Human Ultraconserved Region Reveals Somatic and Constitutional Genomic Instability
Source: PLoS Biol. 2010 Jan 5;8(1):e1000275. doi: 10.1371/journal.pbio.1000275 (PMC2794366; doi:10.1371/journal.pbio.1000275)
Supplement: Table S7 — Comparison of substitution frequency and mutability outside and inside UCR41 after filtering for sequencing errors. Reported is the number of positions with low substitution frequency (<0.1%) outside and inside UCR41 for each sample, after three different filters for sequencing errors were applied. After each filtering, the usual statistical analyses were applied. In particular, the distributions of substitution frequency outside and inside UCR41 were compared using the Wilcoxon test, whereas the observed mutability ratio was compared to the expected distribution after 1,000,000 random permutations (see main text). *Two-tailed Wilcoxon test (alpha value = 0.05). **Probability of observing a mutability ratio equal or higher than the observed value, after 1,000,000 random permutations. (0.06 MB DOC) [file pbio.1000275.s010.doc]

**Table S7:** Comparison of Substitution Frequency and Mutability Outside and Inside UCR41 After Filtering for Sequencing Errors

| **Sample** | **Homopolymers (n>3)** | | | | **Uncalled Reads** | | | | **Positions With Only**  **One Mutated Read** | | | |
| --- | --- | --- | --- | --- | --- | --- | --- | --- | --- | --- | --- | --- |
|  | **Positions** | | **P-value*** | **P**** | **Positions** | | **P-value*** | **P**** | **Positions** | | **P-value*** | **P**** |
|  | **Outside** | **Inside** |  |  | **Outside** | **Inside** |  |  | **Outside** | **Inside** |  |  |
| CC | 886 | 163 | 2x10^-8^ | <10^-6^ | 1030 | 176 | 4x10^-7^ | <10^-6^ | 866 | 131 | 1x10^-4^ | 5x10^-6^ |
| NC | 891 | 165 | 2x10^-4^ | 9x10^-5^ | 1028 | 175 | 7x10^-5^ | 3x10^-5^ | 834 | 145 | 2x10^-6^ | 2x10^-6^ |
| PBL | 842 | 158 | 1x10^-5^ | 6x10^-6^ | 937 | 159 | 7x10^-6^ | 9x10^-6^ | 971 | 128 | 6x10^-5^ | 7x10^-6^ |
| H-PBL | 851 | 156 | 0.23 | 0.17 | 956 | 161 | 0.15 | 0.11 | 823 | 134 | 0.56 | 0.33 |
