# Supplementary material for: Ultradeep Sequencing of a Human Ultraconserved Region Reveals Somatic and Constitutional Genomic Instability
Source: PLoS Biol. 2010 Jan 5;8(1):e1000275. doi: 10.1371/journal.pbio.1000275 (PMC2794366; doi:10.1371/journal.pbio.1000275)
Supplement: Table S8 — Estimation of PCR errors. For each sample, the total number of estimated PCR errors was derived using the binomial probability distribution. Comparable numbers were obtained using the simulation model (see text). The corresponding percentage of PCR errors over the total low-frequency substitutions (<0.1%) was calculated for raw data and after filtering for potential sequencing errors. As expected, the percentage of PCR errors increases after filtering for sequencing errors, since the contribution of errors introduced by 454 sequencing decreases. For observed and expected distributions of mutability ratios, the mean, as well as 95% confidence interval (in brackets), are reported. In each sample, observed and expected distributions were compared using the Wilcoxon test. (0.05 MB DOC) [file pbio.1000275.s011.doc]

**Table S8.** Estimation of PCR Errors

| **Sample** | **Estimated PCR Errors** | **Raw Data** | | | | **Data Filtered for Potential Sequencing Errors** | | | |
| --- | --- | --- | --- | --- | --- | --- | --- | --- | --- |
|  |  | **Percentage of PCR Errors** | **Observed Mutability Ratio** | **Expected Mutability Ratio** | **P-value** | **Percentage of PCR Errors** | **Observed Mutability Ratio** | **Expected Mutability Ratio** | **P-value** |
| CC | 915 | 13.5% | 1.36 (1.29-1.44) | 1.03 (0.87-1.21) | <2x10^-16^ | 17.0% | 1.27 (1.18-1.37) | 1.02 (0.85-1.22) | <2x10^-16^ |
| NC | 847 | 13.5% | 1.17 (1.11-1.23) | 1.02 (0.88-1.18) | <2x10^-16^ | 17.2% | 1.18 (1.11-1.27) | 1.02 (0.86-119) | <2x10^-16^ |
| PBL | 981 | 15.2% | 1.26 (1.19-1.35) | 1.05 (0.89-1.21) | <2x10^-16^ | 19.5% | 1.21 (1.13-1.32) | 1.02 (0.86-1.20) | <2x10^-16^ |
| H-PBL | 901 | 12.2% | 0.95 (0.89-1.01) | 1.03 (0.89-1.18) | 1 | 16.0% | 0.94 (0.88-1.02) | 1.00 (0.86-1.17) | 1 |
